# Preparation and characterization of sodium alginate–PVA polymeric scaffolds by electrospinning method for skin tissue engineering applications

**DOI:** 10.1039/d1ra04176b

**Published:** 2021-09-15

**Authors:** Sorour Jadbabaei, Majid Kolahdoozan, Farid Naeimi, Hassan Ebadi-Dehaghani

**Affiliations:** Department of Chemistry, Shahreza Branch, Islamic Azad University Shahreza Isfahan 31-86145 Iran kolahdoozan@iaush.ac.ir; Advanced Materials Research Center, Materials Engineering Department, Najafabad Branch, Islamic Azad University Najafabad Iran; Department of Chemical Engineering, Shahreza Branch, Islamic Azad University Shahreza Isfahan 31-86145 Iran

## Abstract

Sodium alginate (SA) has proven its high potential in tissue engineering and regenerative medicine. One of the main weaknesses of this polysaccharide is its low spinnability. Nanofiber-based scaffolds are of interest to scientists for biomedical engineering. The main aim of this study was to improve the spinnability of SA in combination with polyvinyl alcohol (PVA). The main parameters in the electrospinning of the optimized SA:PVA ratio, including voltage, flow rate, and working space were also optimized. To achieve this, response surface methodology under central composite design was employed to design the experiments scientifically. The final nanofiber scaffolds were studied using scanning electron microscopy, Fourier transform infrared spectroscopy for degradability, swelling, tensile strength, porosity, nanofiber diameter, contact angle, and cytotoxicity. Based on the results, the best ratio for SA : PVA was 1 : 6.5 that was spinnable in various values for the process parameters. The fabricated scaffolds under these conditions revealed good physical, chemical, mechanical, and biological features. L929 cell lines revealed high viability during 48 h culture. The results revealed that uniform and homogeneous nanofibers with regular size distribution (166 nm) were obtained at 30 kV, 0.55 μL h^−1^, and 12.50 cm. To sum up, the fabricated scaffolds with the optimized ratio under the reported conditions indicate at good biologically compatible candidates for skin tissue engineering.

## Abstract 2

The main aim of this study was to improve the spinnability of SA by adding PVA. The main parameters in producing nanofiber from the optimized SA:PVA ratio, including voltage, flow rate, and working space, were also optimized.

## Graphical abstract

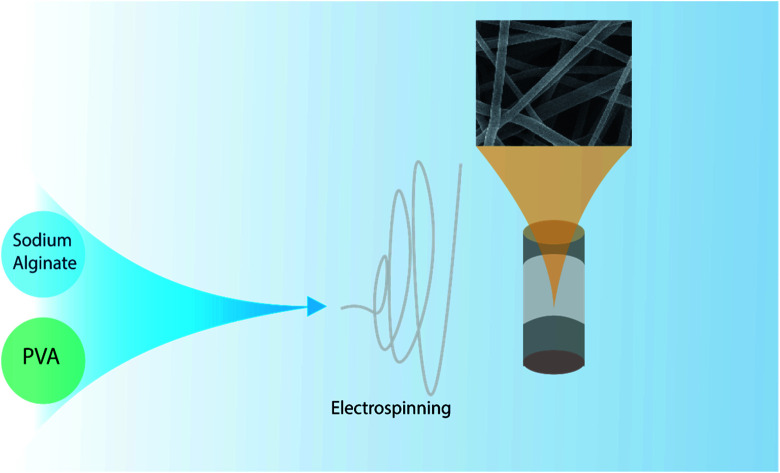

## 1. Introduction

Tissue engineering (TE) is growing as a novel biomedical engineering area to redevelop newfound materials for substituting problematic or injured tissues.^1,2^ It comprises the construction of natural and/or synthetic structures, allowing the combination of these materials with growth factors and/or signaling molecules to modulate cell proliferation and differentiation, and develop constructs mimicking the extracellular matrix (ECM).^3^

The TE of skin substitutes signifies a potential foundation of improved treatment in fighting acute and chronic skin injuries.^4^ Human skin is the widest organ of the body affected by injuries such as infection, burns, and diseases.^5^ There are no significant prototypes of engineered skin that duplicate the composition, structure, organic constancy, or visual environment of healthy skin. Skin alternates should carry some essential physiognomies that are simple to use.^6^

Recent advances in skin TE have offered the potential to improve skin regeneration's clinical outcome.^7,8^ However, some deficiencies need to be addressed to provide substitutes with painless and rapid healing processes and encourage vascular, neural, and lymphatic networks, hair follicles, sebaceous, and sweat glands.^9^ Therefore, skin TE's ultimate goal is to fabricate a complicated scar-free skin substitute that can be transplanted in large quantities in only one surgical intervention with a minimum chance of rejection by the host's body.^10,11^

One of the main factors that influence graft success is the scaffolding technique. Some of the main criteria for designing a scaffold are cell adhesion, infiltration, proliferation, and differentiation, and capability to create new tissue.^12^ Various techniques have been reported for skin TE, including 3D printing, electrospinning, freeze-drying, and gas foaming. Scaffolds fabricated by electrospinning have been classified as an optimal scaffolding option with beneficial biological and mechanical properties.^13,14^ Electrospun nanofibers have exceptional properties such as a structure similar to the natural extracellular matrix (ECM),^15^ permeability,^16^ and scar formation regulation.^17^ This technique has been used extensively in the field of skin TE and various natural and synthetic biomaterials such as polycaprolactone (PCL),^18^ poly (lacto-*co*-glycolic acid) (PLGA),^19^ polyvinyl alcohol (PVA),^20^ sodium alginate (SA),^21^ bacterial cellulose,^22^ chitosan,^23^ and collagen^24^ have been utilized to fabricate electrospun scaffolds (nicely reviewed by Quynh P. Pham *et al.*^25^).

There are several studies considering blends of SA and PVA for TE purposes. In research by Manikandan *et al.*,^26^ they indicated that the SA/PVA composition can be a suitable candidate for liver TE as liver cells had excellent adhesion. In the case of bone TE, SA/PVA 3D printed scaffolds revealed its high potential in cell viability as it possessed homogeneous porosity and improved hydrophilic properties. The scaffold had excellent mechanical properties, and its elasticity showed promising results.^27^ Similarity, Coelho *et al.*^28^ showed that among many polymer-based scaffolds fabricated for TE engineering, SA/PVA scaffolds are known to provide mechanical stability (high tensile strength and elongation at break), and slow degradation kinetics to the scaffolds. Also, Alhosseini *et al.*^29^ showed that in neural tissue, scaffold microstructure, its three-dimensionality, and fiber alignment are as essential as its biological properties. Even though many materials and techniques have been employed in TE, SA/PVA-based electrospun nanofibers have been shown to meet all the requirements. They can be tuned to fit specific alignments, porosity, and architectures while maintaining their flexibility, mechanical properties, and biological features. In research by Vig *et al.*,^30^ SA/PVA blend was used to fabricate an electrospun scaffold for skin regeneration. The fabricated scaffold revealed good mechanical properties, hydrophilicity, cell attachment, and cell growth. In another research in skin regeneration, SA/PVA scaffold showed improved active substance delivery properties in the presence of SA inside the cross-linked polymeric network.^30^

According to previous studies, based on SA:PVA ratio, the SA/PVA scaffold can be applied for both hard and soft tissues. Enhancement in SA content makes the scaffold suitable for soft tissues while increasing the PVA content makes it eligible for hard tissues. Thereby, it is hypothesized that this blend can also be nominated for skin TE. Hence, the main aim of this study was to increase SA electrification capability using PVA to fabricate a new electrospun SA/PVA scaffold capable of supporting skin fibroblasts for skin TE.

## 2 Materials & methods

### 2.1. Chemicals

Sodium alginate (SA, Sigma-Aldrich Canada Ltd, with a molecular weight of 216.12 g mol^−1^) and polyvinyl alcohol (PVA, 99%, Merck), and glutaraldehyde were purchased from a local supplier, TemadKala Co., Tehran, Iran. All the materials and reagents used were of analytical grade.

### 2.2. Procedure

In this work, we tried to fabricate an electrospun SA-based scaffold by optimization of the final formula and the main parameters in electrospinning including flow rate (*Q*), working space (distance from the needle tip to the collector)s (*X*), and voltage (*V*) and performed characterization. To accomplish this, in step#1, first, the optimized formulation of SA and PVA was determined. Then, in step#2, the optimized formula was employed to evaluate the optimized conditions.

### 2.3. Design Expert(DOE)

In this study, response surface methodology (RSM) using central composite design (CCD) was employed to find the optimum formulation to prepare electrospun SA/PVA scaffold with proper fiber diameter, appropriate tensile strength, and high cell compatibility. The main parameters including *Q*, *V*, and *X* were evaluated for optimizing the formulation. Accordingly, the percentage of PVA and SA in bioink composition were considered as the process parameters in DOE. Three levels, including low (−1), medium (0), and high (+1), were defined for PVA and SA concentration separately. According to our literature study, for PVA, low and high levels were 1% and 12% w/w and for SA were 1% and 4%, respectively. As shown in Table 1, 13 runs were performed. Nanofiber producibility was measured as the response. The measured response was transferred in the software, which provided an equation and relevant graphs to show the governed relation between material composition and the considered response. The main aim of DOE was to find out the most optimal condition and composition for making the scaffold.

**Table 1:** Experimental design parameters and responses for SA/PVA electrospinning evaluation

Runs	Coded runs		Factors		Response
	PVA	SA	PVA wt%	SA wt%	Nanofiber producibility
1	−1	0	1.00	2.50	1
2	−1	1	1.00	4.00	2
3	1	−1	12.00	1.00	1
4	0	−1	6.50	1.00	4
5	−1	−1	1.00	1.00	1
6	0	0	6.50	2.50	3
7	0	1	6.50	4.00	2
8	1	1	12.00	4.00	1
9	0	0	6.50	2.50	3
10	1	0	12.00	2.50	1
11	0	0	6.50	2.50	3
12	0	0	6.50	2.50	3
13	0	0	6.50	2.50	3

### 2.4. Polymeric solution preparation

To prepare the polymer solutions accurately, since both SA and PVA are water-soluble, deionized water was used as the solvent. First, the required amount of each substance was weighed according to the DOE results and then transferred to a 50 mL test tube and increased to a volume of 20 mL using deionized water. The tube was placed on a stirrer and the resulting solution was mixed for 12 h. The final solution was sonicated at 170 watts in an ultrasonic bath for 10 min. Finally, the samples were stored in the refrigerator (4 °C).

### 2.5. Electrospinning

Each sample was sonicated for 10 min before starting the electrospinning process. Then, 5 mL of each sample was transferred into a 10 mL syringe. It was noted that the solution was free of any bubbles. The drums were covered by aluminum foil. The electrospinning process was investigated by changing three parameters, including voltage (<30 kV), flow rate (<1 mL h^−1^), and nozzle distance (<30 cm) from the drum.

### 2.6. Crosslinking

Since both polymers PVA and SA are water-soluble, after the fiber production process and drying, this solubility is still high, and on the first contact with the aqueous medium, the fibers dissolve in water (culture medium). The crosslinking process was carried out to improve this issue. In this regard, 25% glutaraldehyde solution was used. For this purpose, the desired pieces were cut from foil and placed in a Petri dish. 2 mL of 25% glutaraldehyde solution was poured into a small container and transferred to Petri dish containing fiber pieces. The Petri dish was sealed with parafilm and was placed in an incubator (Shimaz, Iran) at 37.5 °C for 24 h. At the end of the course, all glutaraldehyde solution was evaporated. This temperature helps glutaraldehyde to evaporate and penetrate through the scaffold matrix under uniform conditions.

### 2.7. Morphological characterization by SEM

To measure the size distribution and surface structure of the 3D printed scaffolds, and cell attachment, scanning electron microscopy (SEM) (Philips XL30; Philips, Eindhoven, Netherlands) was carried out under a 25 kV accelerating voltage after sputtering a 5 nm diameter gold layer on samples. The average strand diameter was calculated using the ImageJ software (National Institute of Health, USA).

### 2.8. Structural characterization by FTIR

To ensure the link between the SA and PVA functional groups and also the chemical bonds, specific values of each sample were prepared and analyzed by an infrared spectrometer (FTIR, SHIMADZU, 8400S model Japan) with KRS-5PRISM at a 45-degree angle. The IR spectrum was recorded in the frequency range 500 to 4000 cm^−1^.

### 2.9. Degradation characterization by weight determination

Scaffolds were freeze-dried and then weighed to determine their initial masses. The samples were incubated in 10 mM phosphate-buffered saline (PBS) solution of pH = 7.40 at 37 °C and 5% carbon dioxide (according to the cell culture conditions) for 3,7, 14, and 21 days to study sample stability in simulated physiological solutions. The PBS solution was removed from samples and then samples were washed with deionized water twice, and then samples were freeze-dried and weighed again using a digital scale. The scaffold degradation was calculated using eqn (1):1
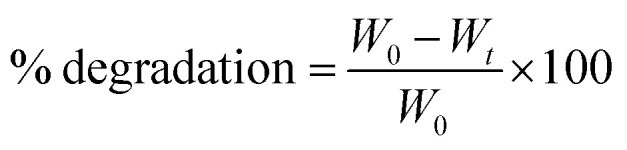
*W*_*t*_ is the freeze-dried scaffold weight at a given time, and *W*_0_ is the freeze-dried scaffold weight at the time zero.

### 2.10. Swelling

The primary weight of scaffolds was measured after cross-linking. The scaffolds were then incubated in 10 mM PBS solution in pH 7.4 at 37 °C and 5% carbon dioxide (according to the cell culture conditions). The samples' weights were measured again after 24 h for any mass change due to swelling. A Kimwipe was used to eliminate excess or free liquid from the scaffolds before weighing each sample. The swelling of the composite scaffolds was calculated using eqn (2):2
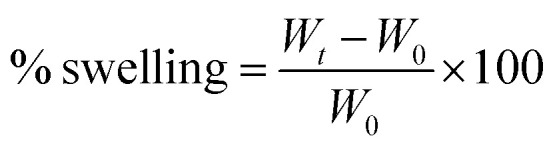


### 2.11. Hydrophilicity characterization by contact angle

To determine and compare the hydrophilicity of different scaffolds, and the water contact angle of the samples was measured. For this purpose, first, the sample was placed on a flat surface and then a drop of water was dropped on it with a moving needle. The spherical image of the droplet was transmitted to the monitor by a digital camera and then the contact angle of the droplet with the web surface of the nanofibers was measured.

### 2.12. Porosity

The porosity of scaffolds was measured from SEM images using ImageJ software. To process the images to obtain the total porosity, the total porosity was measured as the sum of the areas between the fibers, expressed as a percentage.

### 2.13. Cytotoxicity

To evaluate the cytotoxicity of the prepared scaffolds, first, the electrospun scaffolds were immersed in 70% ethanol for 24 h. After drying the scaffolds at room temperature, the scaffolds (both sides) were sterilized for 1 h by exposure to UV rays. The scaffolds were then carefully placed on a plate and washed with sterile PBS. Fibroblast L929 cell line obtained from the cell bank from the School of Advanced Technologies in Medicine (Shahid Beheshti University of Medical Sciences, Tehran, Iran) with a density of 2 × 10^3^ mL^−1^ and were placed on scaffolds by a drip method at a rate of 20 μL. Next, the scaffolds were incubated for 48 h at 37 °C and 5% CO_2_. At the end of the period, 10 μL of the MTT labeling reagent at the concentration of 0.5 mg mL^−1^ was added to each well and incubated for 4 h under the same conditions (37 °C and 5% CO_2_). Then, 100 μL of the solubilization solution was added to each well. The samples were incubated at 37 °C and 5% CO_2_ overnight. The purple formazan crystals were checked and the absorbance was measured by an ELISA reader.

## 3 Result and discussion

### 3.1. RSM statistical study to assess the effect of SA:PVA ratio and the operating parameters

In this research work, it was tried to improve the electrospinning potential of SA by combination with PVA and also study the electrospinning main parameters (*V*, *Q*, and *X*) to produce nanofibers with better quality. To achieve this, as can be seen from Table 1, 13 runs were considered according to the RSM study to find out the nanofiber producibility of each formulation of SA and PVA. Table 2, represents 20 different conditions to produce nanofibers from an optimized formula of SA and PVA. The DOE software provided quadratic equations as the governing relations between the percentage of ingredients and the selected response (nanofiber production) were examined *via* ANOVA. Table 3 summarizes the results.

**Table 2:** Experimental design parameters and responses to study the effect of electrospinning parameters (*V*, *X*, and *Q*)^a^

Runs	Coded runs			Factors			Response
	*V*	*X*	*Q*	*V* (kV)	*X* (cm)	*Q* (mL h^−1^)	Nanofiber producibility (1–5)
1	1	0	0	1.00	12.50	0.55	3
2	0	1	0	15.50	20.00	0.55	2
3	0	0	−1	15.50	12.50	0.10	4
4	1	−1	1	30.00	5.00	1.00	1
5	1	1	−1	30.00	20.00	0.10	4
6	1	−1	−1	1.00	5.00	0.10	1
7	0	0	0	15.50	12.50	0.55	2
8	0	0	0	15.50	12.50	0.55	1
9	−1	1	1	1.00	20.00	1.00	1
10	1	1	−1	1.00	20.00	0.10	3
11	0	0	0	15.50	12.50	0.55	1
12	0	−1	0	15.50	5.00	0.55	5
13	1	−1	1	1.00	5.00	1.00	3
14	1	1	1	30.00	20.00	1.00	3
15	1	0	0	30.00	12.50	0.55	3
16	0	0	0	15.5	12.50	0.55	5
17	0	0	0	15.5	12.50	0.55	3
18	0	0	1	15.5	12.50	1.00	3
19	1	−1	−1	30.00	5.00	0.10	4
20	0	0	0	15.50	12.50	0.55	1

a 5: the high potential of fiber producibility and 1: the low potential of producibility.

**Table 3:** The governed equations and the relevant analysis of variance results

Response	The final equation in terms of code factors	*P*-value	*R* ^2^	Adj. *R*^2^	AP
Nanofiber producibility^a^	2.97 − 0.17A − 0.17B − 0.25AB − 1.88A^2^ + 0.12B^2^	0.01	0.83	0.72	6.83
Nanofiber producibility^b^	3.40 + 0.90A + 0.5B −0.10C + 0.75AB − 0.50A^2^ + 0.50B^2^ − 1.50C^2^	0.016	0.79	0.61	6.3

a A: PVA – B: SA.

b A: *V*, B: *X*, C: *Q*.

**Fig. 1:**
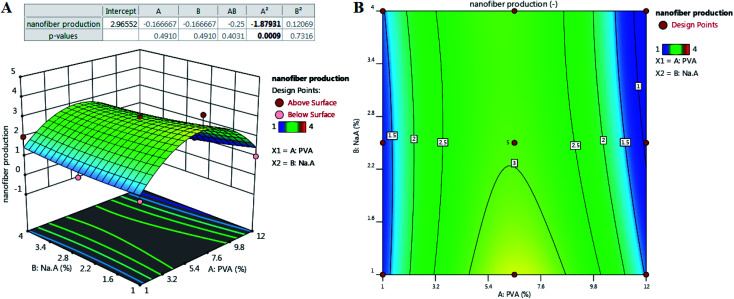
The effect of composition on the nanofiber production: (A) appropriate *P*-value for the main parameters and three-dimensional (3D) surface graph, and (B) counterplot.

The reliability of a model is usually justified *via P*-value, which should be lower than 0.05 to conclude that the model fitting the experimental data are valid and significant.^31^ As can be seen from Table 1, the *P*-value was lower than 0.05 in both studies. Considering the effect of the percentage of SA and PVA on nanofiber producibility, *P*-value was higher than 0.05 for A and B (as the first-order effects), AB (interaction effect), and B^2^ (as the second-order effects). The *P*-value was lower for A^2^ as the second-order effect of PVA (Fig. 1). Regarding the effect of operation parameters, the *P*-value has been reported lower than 0.05, which depicts the validity and significance of the governed equation. The *P*-value was lower than 0.05 only for A as the first-order effect, AB as the interaction order, C^2^ as the second-order effect. The *P*-value was too high for AC and BC and C.

The reliability of a fitted model is specified by the determinant coefficient (*R*^2^) and Adj. *R*^2^ as its adjusted form. The validity of the model can be approved if *R*^2^ ≥ 0.60.^32^ Both models showed *R*^2^ equal to 0.83 and 0.79 and had a reasonable agreement with Adj. *R*^2^, indicating that the models are capable to analyze and predict the response over the change in the process parameters. Adequate precision (AP) compares the range of the predicted values at the design points to the average prediction error, where a ratio higher than 4 is desirable.^33^ As Table 3 depicts, in both models, AP values reported are higher than 4, showing that there was a good agreement between the predicted and experimental values including most of the responses.

Fig. 1 shows the relation of response with the effect of the SA and PVA combination. According to contour results, more of their compounds fail to produce nanofibers and the probability of producing nanofibers is very low. According to the results, the only ratio that showed spinnability was 1 : 6.5. In other cases, no nanofiber was produced due to high or low viscosity and lack of enough surface tension. SA did not show spinnability when employed alone. The reason might be related to the limited solubility and high viscosity of this natural polyelectrolytic polymer. Previous studies have reported that the combination of SA with other polymers increases the spinnability of SA.^34,35^ Due to the formed hydrogen bonds between SA and other polymers such as PCL, the repulsive force between the polyionic molecules is notably reduced to boost the chain fusion, which ultimately leads to the production of nanofibers.^36^ For instance, Gong and his colleagues produced SA-based nanofibers by employing polyethylene oxide (PEO).^33^ Lu *et al.*^37^ studied the electrospinning ability of SA in combination with PEO at a concentration of 1 to 4%. They showed that only 3% of PEO resulted in a smooth and uniform nanofibers. The final viscosity has been reported to play a critical role in spinnability.^38^ In some cases, the addition of surfactants such as Triton-X100 can improve the viscosity and also spinnability.^39^ Based on the obtained governed equation in Table 3, it seems that the addition of PVA in each concentration did not guarantee the spinnability of SA and only the 6.5% of PVA combined with 1% of SA (as the optimized SA:PVA ratio) resulted in nanofibers. To analyze the nanofiber and also the effect of process parameters on the quality of the synthesized nanofibers, this ratio was used in the main formulation for the rest of the study. Other ratios did not result in nanofibers under any adjustment of operating parameters including voltage (0–30 kV), working distance (5–20 cm), and flow rate (0.10–1.00 μL h^−1^).

**Fig. 2:**
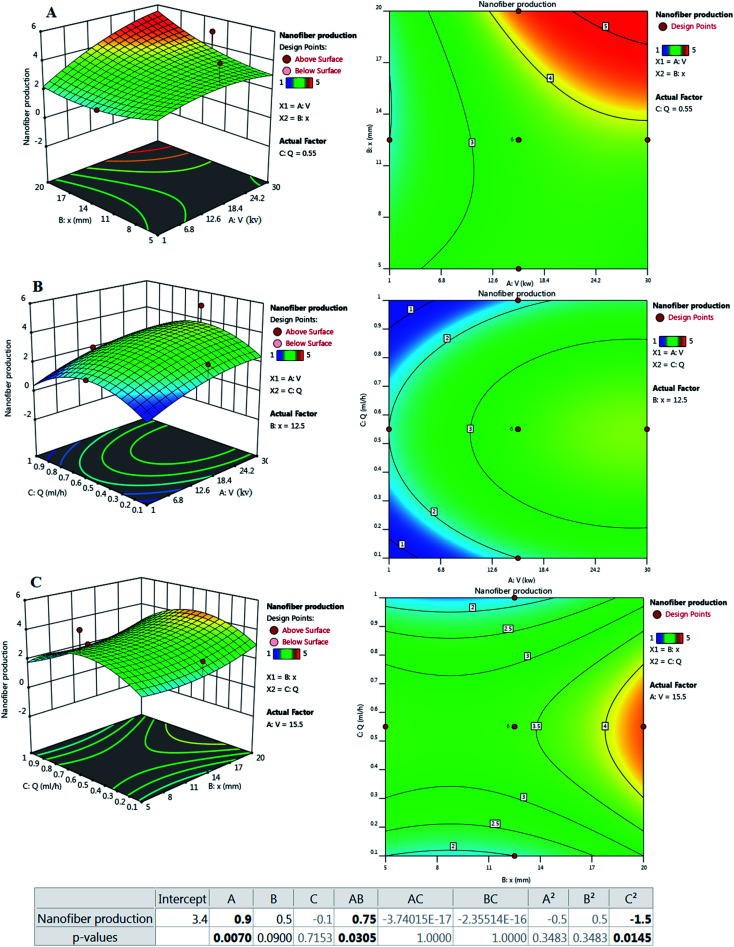
Effect of process parameters on nanofiber production: (left) three-dimensional (3D) surface and (right) counterplot. (A) *V*–*X*, (B) *V*–*Q*, and (C) *X*–*Q*.

Fig. 2 illustrates the relationship between the response and voltage (*V*), distance (*X*), and flow rate (*Q*) as the main parameters in electrospinning of the optimized formulation according to Table 2.

#### 3.1.1 The effect of *X* and *V*

As can be seen in Fig. 2A, at constant *Q*, low *V* affects nanofiber production negatively. To produce nanofibers at low voltage, it is necessary to reduce the working distance (lower *X*). However, it is possible to produce nanofibers at higher voltages and working distances compared to lower levels.

#### 3.1.2 The effect of *V* and *Q*

At constant *X*, as can be seen in Fig. 2B, optimizing *Q*, the probability of producing nanofibers can be increased. Fig. 2B also shows that as *V* increases, *Q* must be adjusted to the medium flow velocity, meaning that at *Q* or higher or lower (set in range) the rotational capacity decreases. However, the distance needs to be adjusted (Fig. 2A).

#### 3.1.3 The effect of *Q* and *X*

It can be seen from Fig. 2C that adjusting *Q* at high or low rates (at constant *V*) cannot lead to the production of nanofibers. The proper *Q* seems to be set at about 0.5 mL per hour but at long working intervals. Under this condition, spinnability is more improved.

In general, *V*, *Q*, and *X* need to be adjusted to increase spinnability. Based on the results, the central points for the values of *V*, *Q*, and *X* appear to be appropriate levels. The applied voltage is a critical factor in electrospinning to generate fibers because the production of nanofibers occurs only when the applied voltage exceeds the threshold voltage.^40^ In the case of voltage, values equal to or above 15.5 kV showed better improvement. In similar studies, a voltage between 12.50–24.00 kV was reported as a suitable voltage for the production of SA/PEO nanofibers. It was reported that too high or too low V fails spinnability.^41^ According to previous reports, increasing the applied voltage increases the electrostatic force of the polymer solution, which is visible in jet traction, and ultimately reduces the length of the nanofibers.^42^ It has also been reported that the applied voltage changes the quality of nanofibers, thus changing the diameter and morphology of the nanofibers.^43^ Reneker *et al.*^44^ stated that the enhancement of the applied voltage does not affect the fiber diameter of PEO. However, in 2005, Zhang and his colleagues reported that obtaining larger diameter nanofibers needs higher voltages because it causes more polymer ejection.^45^ Interestingly, other scientists have reported that an increase in the applied voltage decreases the nanofiber diameter. Furthermore, numerous beads were formed at higher voltages.^42,46^

Another parameter that affects the control of morphology and diameter of nanofibers is the distance of the nozzle from the collector. To control the evaporation of the polymer solution before the fiber reaches the collector, it is necessary to optimize the distance.^47^ Therefore, in the electrospinning technique, an optimized distance is required. Based on the results, the distance depends more on the applied voltage and the flow rate. Longer distances have been reported to produce thinner fibers^48^ but this claim is true when increasing the distance does not disturb fiber formation and power outages.^49^ Also, beads will appear when they are too close or too far.^43,50^ In a study, it was reported that increasing the working distance caused an increase in diameter.^51^ Because the fibers must have sufficient time to cool to achieve uniform fibers and prevent the fiber from fusion, a shorter distance to the collector can increase the likelihood of the fibers fusion at the joint.

*Q* indicates the flow rate of the polymer solution per unit time, which is known as another factor affecting the quality of fibers. It has been reported that increasing the flow velocity leads to the production of larger fibers. Low flow rates are essential for the production of good quality fibers with uniform diameters.^52^ It has been predicted that nanofiber diameter decreases due to increased charge density at low rates.^42^ It was also reported that with increasing flow rate, there is a continuous increase in the nanofiber diameter.^44^ It is noteworthy that excessive flow rate not only increases the integration of nanofibers but also creates beads in the fiber structure due to the lack of sufficient time for the solvent to evaporate.

Experimental results showed that only the following runs showed good nanofiber producibility: (run number: 2-3-5-7-8-11-12-14-15-16-17-18-20). Amongst them, run numbers of 7-8-11-16-17-20 were considered the repeated runs to evaluate the validity of the experiment and monitoring the errors from the operator. The appearance evaluation (data not provided) and also the SEM analysis of these groups were the same and run 8 was considered the representative of these runs. Hence, the only groups employed in the next analysis were 3-5-7-8-12-14-15-18. In the rest, they are named Scaffold3, Scaffold5, Scaffold7, and so on.

### 3.2. Morphology and physical evaluation of the nominated scaffolds

**Fig. 3:**
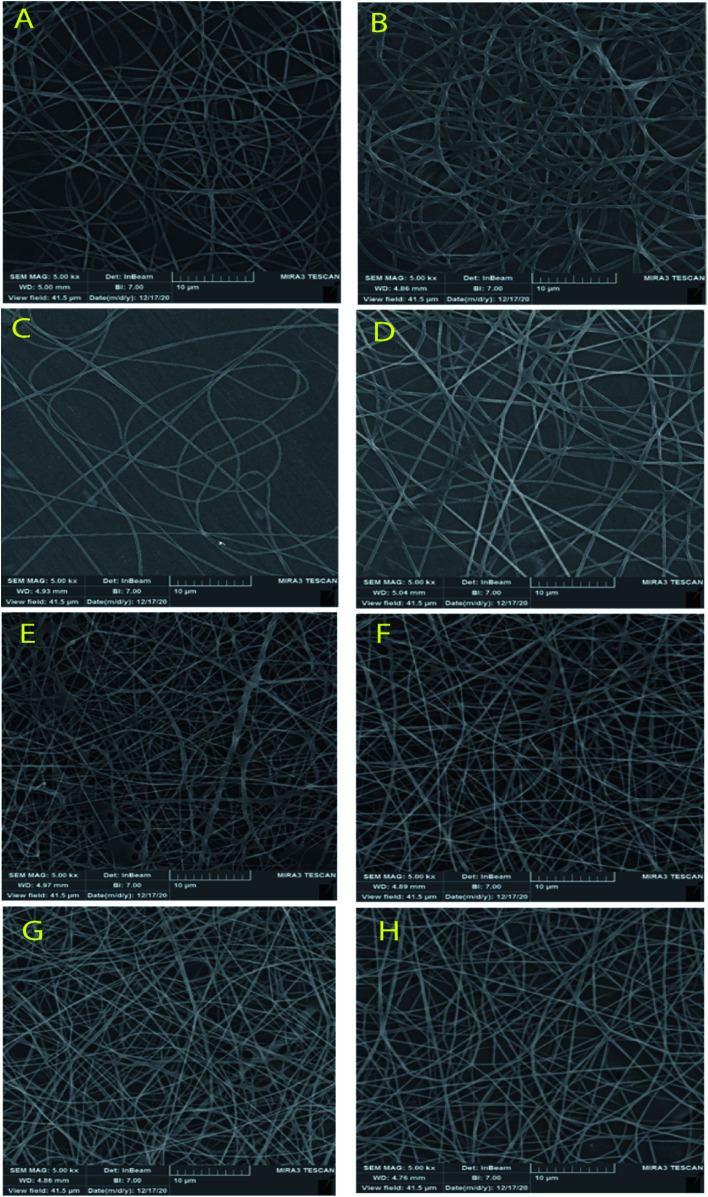
SEM images of the SA/PVA electrospun scaffolds at 10 μm magnitude. (A) Scaffold 2, (B) scaffold3, (C) scaffold5, (D) scaffold7, (E) scaffold12, (F) scaffold14, (G) scaffold15, (H) scaffold18.

**Fig. 4:**
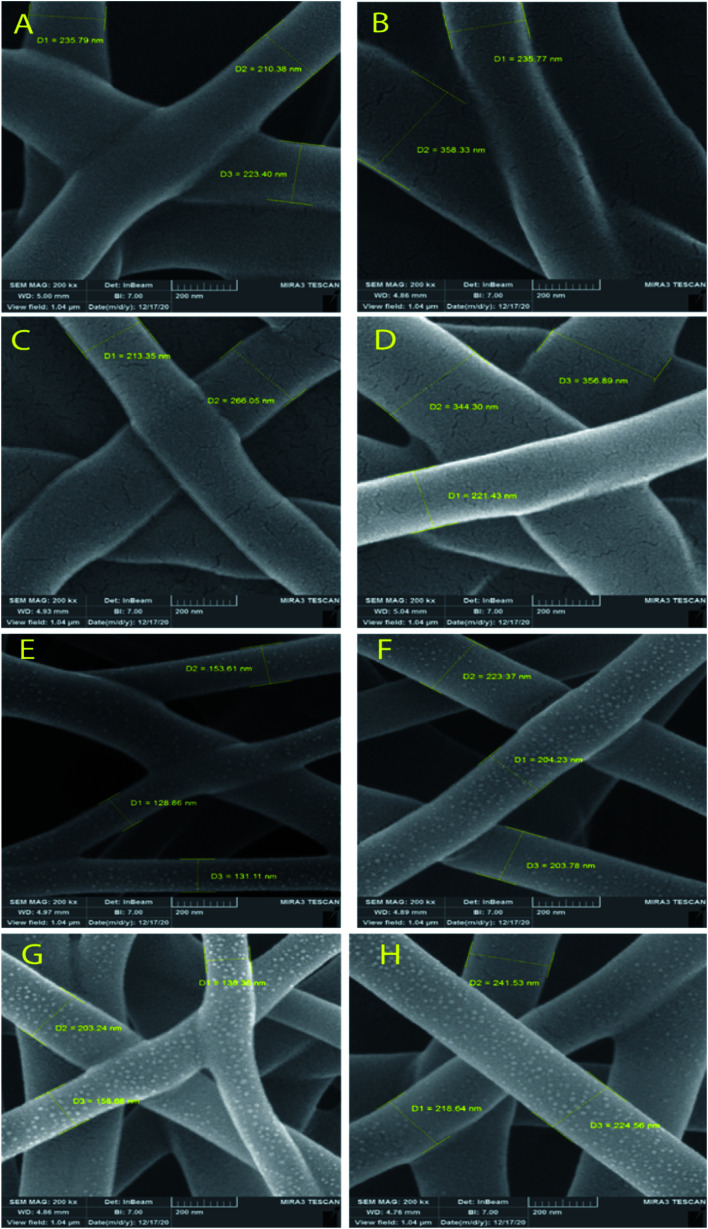
SEM images of SA/PVA electrospun scaffolds for the size distribution at 200 nm magnitude. SA:PVA ratio: (A) scaffold2, (B) scaffold3, (C) scaffold5, (D) scaffold7, (E) scaffold12, (F) scaffold14, (G) scaffold15, (H) scaffold18.

Fig. 3 and 4 show SEM images of the scaffolds with two magnifications and fiber diameter distribution. The porosity and fiber diameters are reported in Table 4. As can be seen, scaffolds showed differences in nanofiber density, distribution, diameter, and electrospinning quality with fewer or no beads. Fig. 3 shows that scaffolds3 and scaffold12 did not have uniform nanofibers in size distribution and quality. The voltage was equal for both scaffolds, but they were different in the distance and flow rate. Considering scaffolds 2, 5 and 7, although nanofibers had uniformity, showed a low density of nanofibers, which could be due to the mismatch of flow velocity with distance. A low flow rate (<0.55 μL h^−1^) and the voltage (>15 kV) can be considered the main reasons for low density. This is while the scaffold14, scaffold15, and scaffold18 illustrated better results in density, uniformity of nanofibers, and smoothness of fibers. According to Table 4, the lowest and highest porosity belonged to scaffold14 (521 nm^2^) and scaffold3 (1404 nm^2^) respectively. Scaffold2 and scaffold7 also had high porosity equal to 1004 nm^2^ and 1205 nm^2^, respectively. Considering the size distribution of nanofibers, the data in Table 4 also show that thin nanofibers belonged to scaffold12 and scaffold15 (140–170 nm) and scaffold3 and scaffold7 showed the thickest nanofibers (300 ± 5 nm). Scaffolds 2, 5, 14, and 18 showed nanofibers in the range of 220–240 nm. It can be hypothesized that applying a higher voltage between 15.5–30.0 kV, adjusting the distance between 12.5–20.0 cm and providing the flow rate at 0.5–1.0 μL h^−1^ resulted in appropriate nanofibers. The results were in agreement with previous studies. Hu and his colleagues produced SA/PEO nanofibers with 120–160 nm diameters under 12, 18, and 24 kV.^41^ Compared with the nanofibers produced in our research, a little difference is observed that may be attributed to the process parameters (140–170 nm v. s 120–160 nm). In another study, the SA/PVA nanofibers were produced with 140–350 nm in diameter under similar conditions (12–30 kV).^53^Table 4 also depicts the results from the contact angle analysis, which was due to the high hydrophilic features of both SA and PVA, the reported contact angle for all scaffolds was lower than 5° meaning that the scaffolds are extremely hydrophilic.

**Table 4:** The physical properties of the selected scaffolds

Scaffold	Porosity area (nm^2^)	Fiber mean diameter (nm)	Contact angle
Scaffold2	1004.67	222.60	<5°
Scaffold3	1404.15	296.50	<5°
Scaffold5	791.83	239.50	<5°
Scaffold7	1205.02	307	<5°
Scaffold12	556.00	137	<5°
Scaffold14	521.32	210	<5°
Scaffold15	619.97	166	<5°
Scaffold18	829.57	227	<5°

According to the results from the morphology analysis and quality evaluation of the synthesized nanofibers, scaffolds 14, 15, and 18 were selected for further analysis. These scaffolds revealed appropriate density, uniform size distribution, and suitable porosity. In the rest of the study, the scaffolds were first cross-linked under 25% glutaraldehyde vapor and then were evaluated.

### 3.3. Chemical structure

**Fig. 5:**
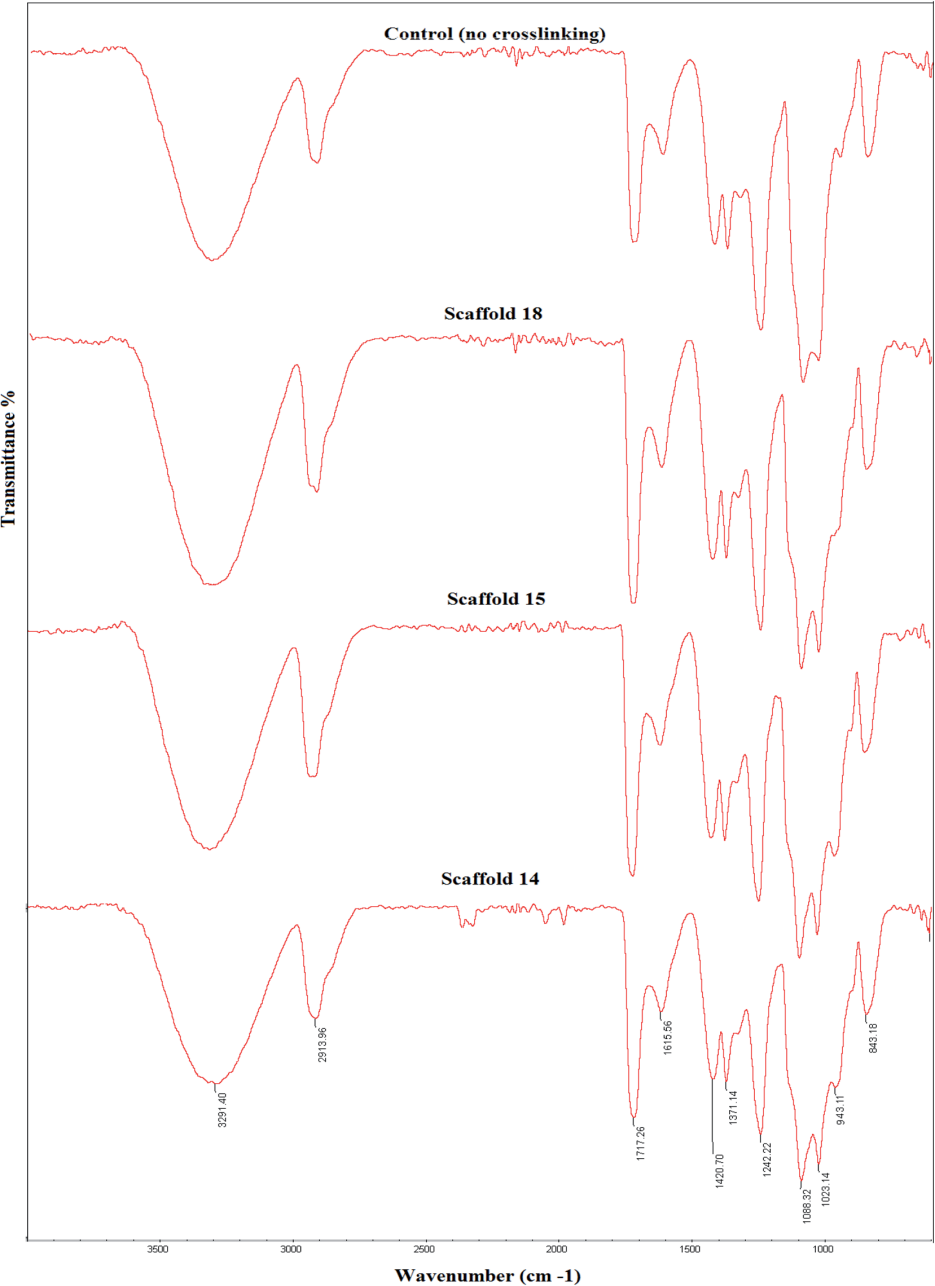
Fourier-transform infrared (FTIR) spectra of the elected electrospun scaffolds and their comparison with the control group, which received no crosslinking.

Fig. 5 shows FTIR spectra of the three selected cross-linked scaffolds (scaffold14, scaffold15, and scaffold18). Since the selected SA:PVA ratio was the same for all scaffolds, as such, one of the scaffolds without crosslinking was nominated as the control group. The SA/PVA electrospun scaffolds showed peaks in the same areas. The characteristic bands for SA were in the range of 3600 and 1500 cm^−1^. The characteristic bands of scaffolds spectrum (Fig. 5) are as follows. Peaks appearing at 3291 cm^−1^ and 2913 cm^−1^ belonged to O–H stretching (hydroxyl group) and C–H stretching vibration, respectively.^54^ The peak at 1088 cm^−1^ belongs to the CN group. The sharp peak at 1717 cm^−1^ is attributed to the carboxylate group.^55^ Compared with the control group, a shoulder before the peak at 1087 cm^−1^ was observed that expanded the peak. Besides, a new peak at 943 cm^−1^ belonging to the CH_2_-rocking vibration,^56^ which can probably be attributed to the process of crosslinking by glutaraldehyde.^57^ The spectra of scaffolds were similar to that of pure PVA^58^ that the reason might be the high PVA content of all samples (SA : PVA; 1 : 6.5). The peak at 843 cm^−1^ is attributed to C–C stretching.^59^

### 3.4. Degradation

**Fig. 6:**
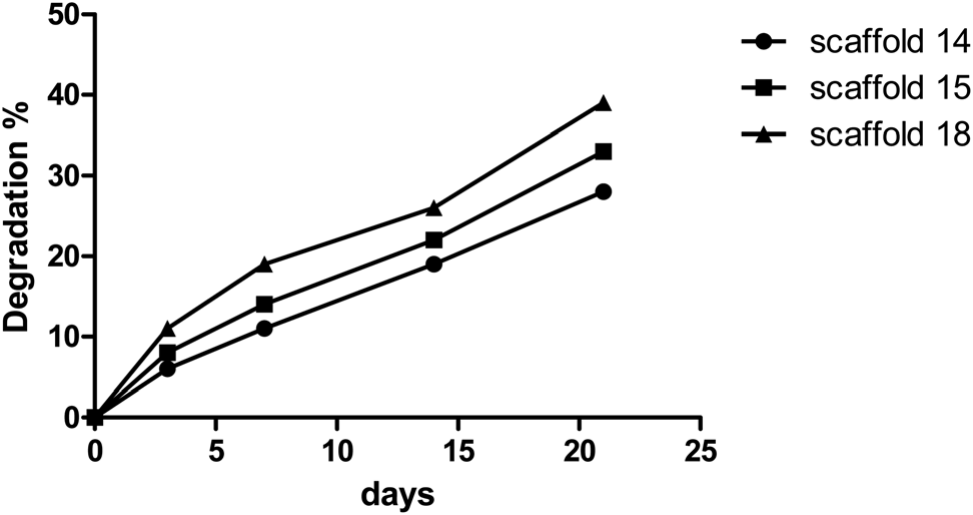
Degradation rate of the selected electrospun scaffolds.

The degree of degradation of each scaffold was also measured by observing a change in the mass of the samples after immersion in PBS over time. Fig. 6 depicts the degradation behavior of the scaffolds during incubation. Scaffold14, scaffold15, and scaffold18 showed 28%, 33%, and 39% degradation, respectively, after 21 days of incubation in PBS with similar patterns. The low and high rates of degradation belonged to scaffold14 and scaffold18, respectively. Various reasons can influence the degradation behavior. Although the scaffolds experienced the same conditions in the cross-linking process, there is a possibility of differences in the level of crosslinking. However, the results from FTIR did not show significant differences in crosslinking and chemical structures. Lim *et al.*^60^ reported that crosslinker and the time of crosslinking could affect the degradation process. The changes in the electrospinning parameters lead to a difference in the density and diameter of nanofibers. The greater the number of nanofibers, the more chemical band there is between the polymer chains.^61^ Based on the results of porosity and SEM, it is hypothesized that higher porosity can be considered the vital parameter in the rate of degradation behavior. The higher porosity resulted in low density and crosslinking.^62^

### 3.5. Swelling

The swelling behavior of the scaffold demonstrates the ability of nutrients and wastes to exchange between the environment and cells embedded in the scaffold to produce artificial tissue. Swelling directly refers to the ability to hydrate and stabilize within biological systems.^63,64^ All scaffolds were incubated in PBS to evaluate the rate of water absorption over time.

**Fig. 7:**
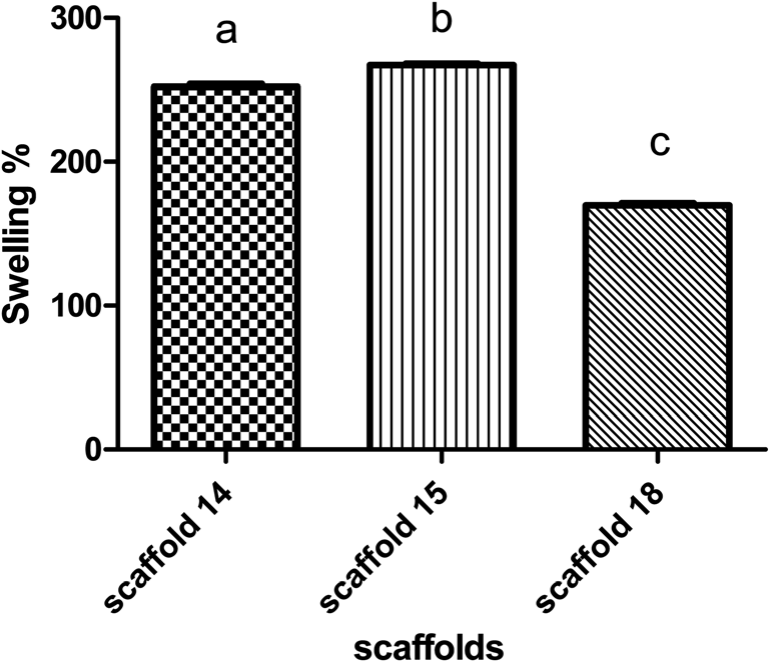
Swelling behavior of the elected electrospun scaffolds.

The behavior of scaffolds in water absorption and swelling showed similar trends (Fig. 7). Scaffold14, scaffold15, and scaffold18 revealed 250%, 260%, and 160% swelling, respectively, after 24 h of incubation. According to the data, scaffolds 14 and 15 showed the highest water absorption in contrast with another scaffold18. This can be due to the high porosity of scaffolding 18, which indicates the low density of nanofibers.^65^ It has been reported that the swelling potential of the scaffolds can be affected by the degree of cross-linking, amorphous regions, and level of hydroxyl groups.^66–68^ According to FTIR results, no significant chemical difference was observed between samples, so it seems that the degree of crosslinking did not affect swelling notably. Comparing all scaffolds, they are made of an SA/PVA compound while there were differences in operating parameters. The nanofiber diameter is one of the vital parameters of electrospun scaffolds and is affected by surface tension, solution viscosity, working distance, flow rate, crystallization characteristics, and applied voltage.^69^ The nanofiber diameter can also affect the porosity of the scaffold, thereby it could be concluded that operating conditions can alter the level of porosity.^12^ This effect may appear in the density of nanofibers per 1 cm^2^ and the diameter of nanofibers (166–227 nm).

### 3.6. Tensile strength

**Fig. 8:**
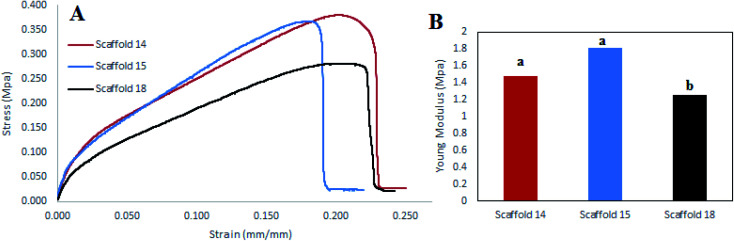
(A) Stress–strain diagram, (B) elastic modulus diagram for scaffold14, scaffold15, and scaffold18.

Tensile strength is a critical factor in studying the mechanical behavior of a scaffold. The tensile strength (MPa) of the scaffolds was measured by determining the strain–stress curve and measurement of the elastic modulus (EM) of each scaffold (Fig. 8). Scaffold14 and scaffold15 had closed trends (Fig. 8A) indicating no significant difference (*P* > 0.05) in EM than the scaffold18 (Fig. 8B). However, scaffold15 revealed higher EM compared with scaffold14 and scaffold18. Scaffold18 showed the lowest EM (*P* < 0.05) (Fig. 8B). It has been approved that high porosity affects the mechanical behavior negatively.^70^ It was also reported that crosslinking can be one of the main factors affecting the mechanical behavior of scaffolds.^71^ Hence, according to Table 4, scaffold18 has higher porosity for which low EM could be predicted. In the viewpoint of nanofiber diameters, interestingly, a reduction in nanofiber diameters caused an enhancement in the mechanical response including Young's modulus and tensile strength, wherein, the superficial limitation of the chains in the distribution of stresses in the fibers was considered the main reason.^69^ It can be hypothesized that nanofibers with uniform distribution of diameter result in a uniform structure that leads to a higher resistance to the axial tensile forces. In this regard, it was reasonable that scaffold15 reveal higher EM.

### 3.7. Cytotoxicity evaluation

**Fig. 9:**
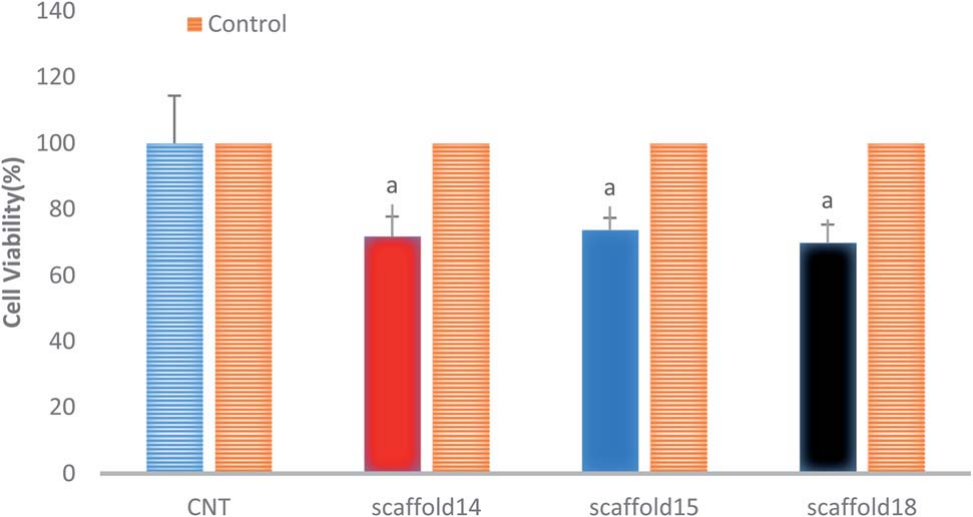
Cell viability analysis of the elected electrospun scaffolds.

This study aimed to fabricate a SA/PVA electrospun scaffold for skin TE, therefore, it was necessary to assess the cytotoxicity and biocompatibility of the scaffolds. The MTT assay was selected for the assessment of scaffolds for fibroblast L929 cell line viability, as shown in Fig. 9. According to the cytotoxic assay, there was no significant difference in viability (*P* > 0.05) between the scaffolds compared with the control group (>75%), which means that all three scaffolds are suitable for cell culture and skin TE purposes. Based on the results of swelling and porosity assessment, variation in porosity and diameter of nanofibers did not make a significant difference in the scaffold cell viability. On the other hand, based on the cell growth assessment as well as cell concentration in each scaffold, it can be claimed that all scaffolds showed good cell adhesion. Previous studies have reported on the biocompatibility of SA, PVA, and SA/PVA. For instance, Wei and You-Lo fabricated SA/PVA hybrid fibers (PV : SA = 40 : 60%) under the physical crosslinking. They reported that the nanofibers were biocompatible and showed no cytotoxicity.^53^ Pure SA also showed higher biocompatibility and higher potential in TE, no cytotoxicity for this polysaccharide has been reported yet.^72^ Regarding PVA, biocompatibility results from previous studies demonstrating that pure PVA was slightly toxic and irritant to the surrounding tissues.^73^ However, it was reported that PVA biocompatibility can be improved when integrated with other biocompatible polymers, including collagen, SA, gelatin, and so on.^74^

## 4 Conclusions

The spinnability of sodium alginate (SA), a biodegradable and biocompatible polymer, was first assessed in combination with different percentages of polyvinyl alcohol (PVA). Then, the optimized SA:PVA ratio was selected to optimize the processing parameters including voltage, working distance, and flow rate. SA inherently is not spinnable, thereby combination with other spinnable polymers improves its potential for nanofiber production. Different percentages of PVA were studied and only the 6.5 PVA depicted good spinnability. The spinnability of the optimized ratio could be controlled with the variation of the applied voltage, flow rate, and working distance. Some operating conditions did not result in nanofibers. The results revealed that the uniform and homogeneous nanofibers with regular size distribution and a narrow diameter (<170 nm) were obtained at 15–30 kV, 0.55–1.00 μL h^−1^, and 12.5–20.0 cm. The fabricated scaffolds under these conditions revealed good physical, chemical, mechanical and biological features.

## Ethics approval

Since we do not receive any funding for this work, thank you for giving us a discount for APC charging.

## Author contributions

This research has been carried out with the collaboration of all authors as below: Majid Kolahdoozan: conception or design of the work, editing paper, paper revision; Sorour Jadbabaei: doing experiments, data collection, writing paper; Farid Naiemib: data analysis and interpretation, paper revision; Hassan Ebadi-Dehaghanic: data analysis and interpretation, paper revision.

## Conflicts of interest

All authors declare no financial/commercial conflicts of interest.

## Supplementary Material
